# Early Intervention in Psychosis services: A systematic review and narrative synthesis of the barriers and facilitators to implementation

**DOI:** 10.1192/j.eurpsy.2021.2260

**Published:** 2021-12-16

**Authors:** N. O’Connell, K. O’Connor, D. McGrath, L. Vagge, D. Mockler, R. Jennings, C. D. Darker

**Affiliations:** 1 Discipline of Public Health and Primary Care, Institute of Population Health, School of Medicine, Trinity College Dublin, Dublin, Ireland; 2 National Clinical Programme for Early Intervention in Psychosis, Health Service Executive Dublin, Ireland; 3 Rise, South Lee Mental Health Services, Cork & Department of Psychiatry, University College Cork; 4 Trinity College Dublin Library, Trinity College Dublin, Dublin, Ireland

**Keywords:** barriers, early intervention, Early intervention in psychosis, facilitators, implementation, psychosis

## Abstract

**Background:**

Early intervention in psychosis (EIP) services target the early manifestation of psychosis and provide multidisciplinary care. They demonstrate effectiveness and cost-effectiveness. Implementation of EIP services is inconsistent and piecemeal. This systematic review and narrative synthesis aims to identify barriers and facilitators to EIP service implementation.

**Methods:**

We conducted an electronic search of databases (EMBASE, Medline, Web of Science, and PsychINFO) to detect papers reporting EIP service implementation findings and associated barriers and facilitators. The search occurred between June to August 2020, and again in January 2021. Articles meeting inclusion criteria were extracted and narratively synthesized. A quality assessment was conducted using the Mixed Methods Appraisal Tool.

**Results:**

Twenty-three studies were selected. The most common study design was descriptive accounts of implementation. Patient age ranged varied from 14 to 35 years. We identified three barrier and facilitator domains: (a) system; (b) services; and (c) staff, and a range of subdomains. The most frequent subdomains were “funding” and “strength of collaboration and communication between EIP and outside groups and services”. Associations between domains and subdomains were evident, particularly between systems and services.

**Conclusions:**

A range of barriers and facilitators to EIP implementation exist. Some of these are generic factors germane across health systems and services, while others are specific to EIP services. A thorough prior understanding of these challenges and enablers are necessary before implementation is attempted. Accounting for these issues within local and national contexts may help predict and increase the likelihood of services’ success, stability, and longevity.

## Introduction

Psychotic disorders can be debilitating [1] and is costly [2]. Their international incidence is 21.4 per 100,000 person years [3]. Outcomes for patients and families are poor [4]. First episode psychosis (FEP) may adversely affect individuals’ educational, employment, and social development through the accumulation of impairment and disability. Greater durations of untreated psychosis (DUP) are moderately associated with worsened prognosis [5]. Early intervention in psychosis (EIP) is associated with positive effects on clinical and functional status at 5-year follow-up in FEP [6], although there are gaps in treatment access [7].

EIP services detect and treat psychotic symptoms early to help stem symptoms and associated behavioral and psychosocial problems. Fidelity scales list objective criteria by which EIP programs can be judged to adhere to sets of standards [8]. Common characteristics of EIP for FEP include early detection, small patient-to-staff ratios, antipsychotic prescription and monitoring, provision of psychosocial and behavioral treatments, 1–3 years program duration, explicit admission criteria and defined missions to serve specific geographic populations. Not all EIP services look the same, but most share some characteristics described in published standards and fidelity scales.

As the EIP evidence base has grown, relatively well-developed services have been implemented in England, Canada, Australia, and Scandinavia. A survey of 29 European Psychiatric member countries reported most countries had 1–5 EIP or early detection services, with 1–2 sites in 38.9% of evaluated countries. Of the 16 countries providing data, duration of services was 15.5 years, with Germany having the longest service duration [9]. Implementation is not widespread, and services are “not yet a broadly accepted or consistent feature of care in most developed countries” [10]. In 2008, the US RAISE program was launched, and within a decade was expected to lead to the establishment of 100 EIP teams [11], yet large-scale implementation has not occurred [12]. Implementation is piecemeal and momentum slow [13].

The implementation gap may be partly due to difficulties in embedding multi-component services within healthcare systems without universal healthcare [14] and higher start-up costs compared with treatment-as-usual. Within Psychiatry, there are debates about EIP’s value, where EIP was viewed as a resource and skill diversion from mainstream services, led by “self-confessed evangelists” [15]. While early intervention is a familiar medical concept, it is novel in mental health services [13]. Equivalency in mental and physical health financing is rare, and services for severe mental illness are subject to political disinterest and stigma [16]. Given this broader context, we hypothesized that implementation success would be linked to the strength, resilience and financing of the existing health and mental healthcare system.

There are likely other implementation challenges. Implementation science attempts to promote the uptake of research findings in real-world settings [17]. Common implementation outcomes include assessment of the adoption of, fidelity to and sustainability of a service or intervention, rather than the intervention’s outcomes [18]. This approach has been successfully applied examine components of complex health systems, for example, research on improving rates of thrombolysis in acute stroke found rates improve with urban location, centralized service models, treatment by neurologists, admission via ambulance, and stroke-specific protocols [19]. A better understanding of these kinds of contextual and human factors, drawn from existing EIP implementation literature, could assist commissioners, policymakers and clinicians in service development. To the best of our knowledge, there are two descriptive reviews [11,20] on the broad status of EIP service implementation, but no systematic review has collated evidence from existing studies on the barriers and facilitators to EIP implementation. Against this background, the current systematic review and narrative synthesis aims to identify the barriers and facilitators to EIP service implementation.

## Methods

A systematic review collated evidence from previous studies of EIP implementation, in accordance with Preferred Reporting Items for Systematic Reviews and Meta-analyses (PRISMA) guidelines [21]. This method was then combined with a narrative synthesis grounded in guidelines developed by Popay et al. [22] to identify and explore the barriers and facilitators of EIP service implementation.

### Registration

This systematic review is registered on PROSPERO (reg no.: CRD42021241603).

### Search strategy

A search strategy was developed with a medical librarian for EMBASE, Medline, Web of Science and PsychINFO databases and conducted between June to August 2020 and again in January 2021. Results were limited to articles published up until January 2021. See “Supplementary Materials” for search strategies applied. Duplicated studies were removed. A secondary hand search of references was performed to identify additional relevant papers in the field.

### Eligibility criteria

This review sought studies reporting data on barriers and facilitators to EIP implementation. Table 1 presents inclusion and exclusion criteria. A study was eligible if it included information on the implementation of EIP services in any jurisdiction at any time. We did not include services for patients with only prodromal symptoms, those with an at-risk mental state only, or high risk or ultra-high risk psychosis only. Studies recording only patient outcomes were excluded, as well as studies which assessed specific EIP service components only (e.g., psychotherapy alone) and which contained no information on service implementation as a whole.

**Table 1.** Inclusion and exclusion criteria applied.

1. Types of studies	Quantitative or qualitative original studies published in full including:
	Interviews/focus groups
	Surveys/questionnaires
	Case studies/service audits/service implementation descriptions
	Feasibility studies/process evaluations
	Systematic reviews/narrative reviews/qualitative meta-syntheses
	Exclusions: Conference papers, papers in languages other than English, oral presentations not available in full text, book chapters, protocols, critique, or theory building papers
2. Study settings	Early intervention in psychosis settings including:
	Community settings
	Mixed context studies (i.e., studies taking place in differing contexts where information on community services specifically is available)
	Exclusions: Inpatient settings
3. Population	Patients attending EIP services including:
	Children or adults
	FEP patients/those experiencing early symptoms of psychosis
	Staff of EIP services of any type
	Exclusions: Services catering to patients with only prodromal symptoms, those with an at-risk mental state only, or high risk or ultra-high risk psychosis (i.e., where the EIP service does not treat patients with an established psychosis diagnosis)
4. Interventions	Any services or interventions which provide EIP services at a local, national or trans-national level where there is an emphasis on assessment, diagnosis, treatment or follow-up of psychosis and where the study provides descriptive, operational or evaluative data on EIP barriers and facilitators to implementation
	Exclusions: services or interventions in other physical or mental health conditions that do not include reference to psychosis
5. Formal collection of data on implementation barriers and facilitators	The study contains formal data (either quantitative or qualitative) from patients, staff or service evaluators on the barriers and facilitators to implementation (at any stage: pre-, post-, or during the process) including studies that provide descriptive or anecdotal information on implementation

Abbreviations: EIP; early intervention in psychosis; FEP, first episode psychosis.

### Study selection process

**Figure 1.**
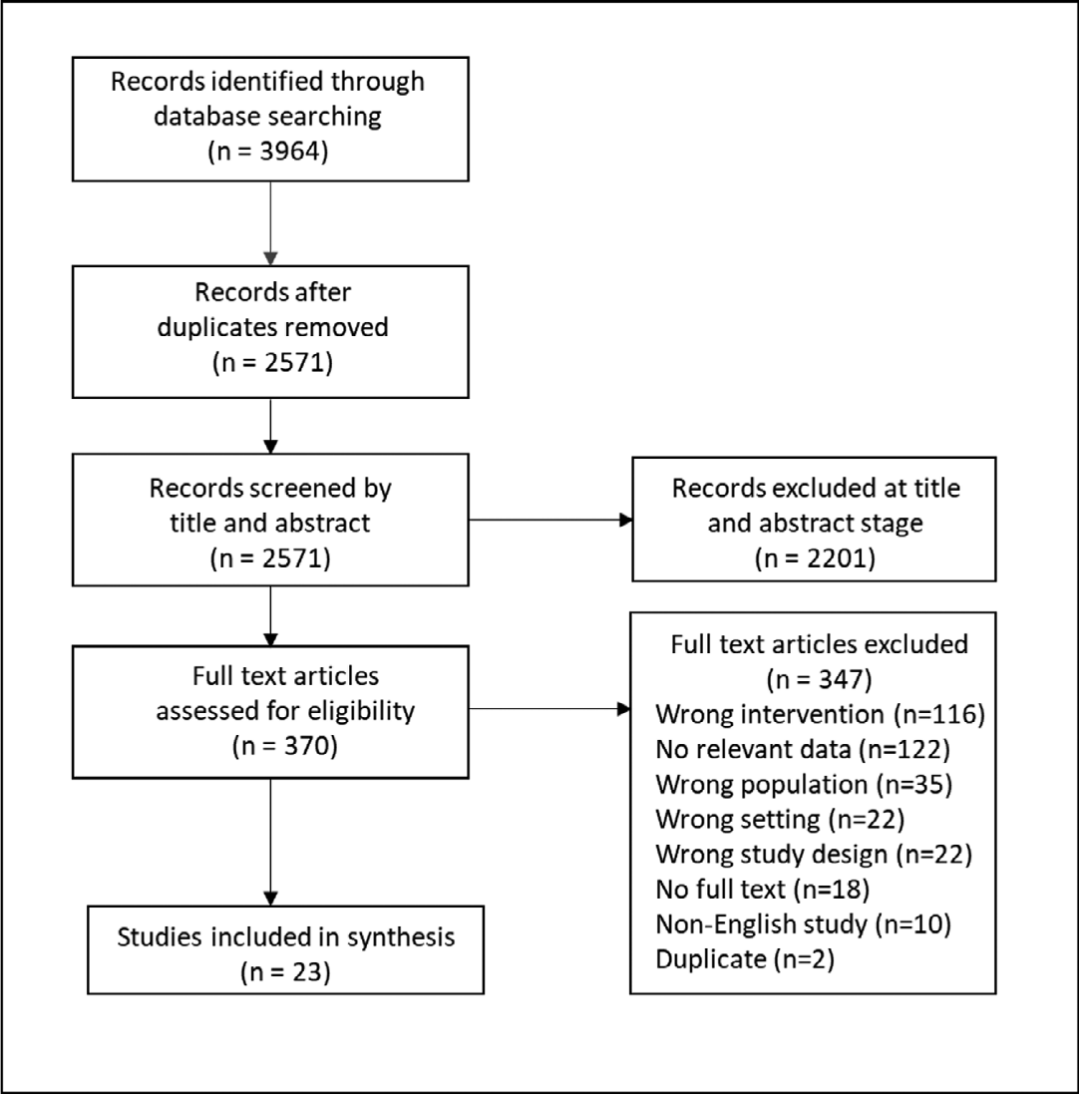
PRISMA flow diagram of study selection process.

Study eligibility was assessed by two authors (L.Z. and D.M.) using Covidence software. L.Z. and D.M. independently screened all titles and abstracts. Disagreements were resolved by discussion, and where necessary, involved a third author (N.O.C.) until consensus was reached. Articles’ full texts were screened by two authors (L.Z. and D.M.) and again, discrepancies were resolved through discussion, and where necessary through involvement by N.O.C. The PRISMA flow diagram (Figure 1) displays search, screening, and selection results.

### Data extraction

NOC performed data extraction using an Excel data extraction form specifically designed for this review. The following quantitative and qualitative information were extracted: (a) studies: authors’ name, year of publication, study country, aims, and methods; (b) participants: participant number and type; (c) EIP services: number and type of sites sampled, type of services, and barriers and facilitators to EIP implementation. To extract data on barriers and facilitators, we generated a data abstraction matrix to organize and display content, an approach developed previously by Geerligs et al. [23].

### Data analysis

The narrative synthesis procedure was derived from Braun and Clarke’s [24] thematic analysis approach, a technique successfully applied in previous synthesizes of health system barriers and facilitators [23,25]. Data analysis was completed in the following stages: (a) reviewing included articles; (b) deriving codes and subcodes that reflected key concepts within the data; (c) developing these concepts into an overarching thematic framework of categories; (d) indexing each article according to the framework and entering summary data into the cells of the abstraction matrix. Initial codes were generated by N.O.C. and further refined to ensure clarity. The results section presents a systematic description of the studies identified, followed the narrative which discusses the themes arising from all studies.

### Quality assessment

Quality assessment was undertaken using Mixed Methods Appraisal Tool (MMAT) [26]. MMAT has sound psychometric properties and allows assessment of quantitative descriptive studies, qualitative and mixed methods studies. Eleven studies were descriptive accounts of EIP implementation and beyond the scope of quality assessment [11,14,27–35]. They were retained as they contained important implementation information. The remaining 12 studies were assessed using MMAT by N.O.C. [36–47]. A subset (*n* = 5) were reviewed by a second author (C.D.) to assess agreement. Agreement was defined as the proportion of items where both raters gave a positive (yes) or a negative (cannot tell, no) score. Agreement analysis was based on Cohen’s Kappa for inter-rater reliability. Scores varied between 0.6 and 1.0, with a total score of 0.8, indicating substantial agreement. Discrepancies were resolved through iterative discussion.

## Results

### Systematic review

#### Included studies

Of 3,964 studies identified, 23 met inclusion criteria. Summary study characteristics and study references are reported in Table 2.

**Table 2.** Summary information displaying author, year, title, country, methodology, and key barriers and facilitators of included studies.

Author and Year	Title	Country	Study method	Key barriers	Key facilitators
Baumann [26]	Treatment and early intervention in psychosis program (TIPP-Lausanne): Implementation of an early intervention program for psychosis in Switzerland	Switzerland	Program description	- Limited funding - Forced to embed initiative into an existing service	- Coherent program - Well-defined focus - Emergence of clinical creativity - Strong academic links
Brabban [42]	What makes early intervention in psychosis services effective? A case study	England	Case study	- High caseloads meaning limited time to deliver interventions - Staff competence and confidence - Delays in staff appointments - Limited program evaluation - Lack of support worker - Virtual location	- Staff training completed before entering EIP service - Increased time per patient - Caseloads capped - Regular clinical supervision - Demonstration of therapeutic optimism and strong philosophy of recovery
Cheng [35]	Matryoshka Project: lessons learned about early intervention in psychosis program development	Canada	Qualitative interviews with staff involved in implementation	- Lack of provisional implementation guidelines for EIP - Embedding of staff into general mental health agencies created overextension of scope of practice and confusion about supervision	- Partnerships and collaboration across sectors and between local and provincial providers - Programs adapted to local conditions, constraints and needs
Cocchi [25]	Early intervention in psychosis: a feasibility study financed by the Italian Center on Control of Maladies	Italy	Feasibility study	- Lack of collaboration between psychiatric services and the potential referrals emerged during the formation of the team - Lack of resources - No incentives for staff beyond initial training	None described
Csillag [10]	Early intervention in psychosis: from clinical intervention to health system implementation	Multicountry	Narrative review	- Lack of political interest - EIP programs driven by academics - Lack of communication with relatives, other professionals, politicians and administrators - Lack or insufficient funding - Obstacles within healthcare systems - Poor coordination with primary care − Poor access to services - Facilities poorly adapted to young people	- Adoption by local authorities of EIP services according to guidelines & clinical evidence - Alignment with service users and family members from service initiation - Services embedded within publicly funded healthcare systems - Central coordination with regional variability in healthcare - Partnership with administrations, fundraising bodies and marketing firms
Durbin [30]	A first step in system improvement: a survey of Early Psychosis Intervention Programs in Ontario	Canada	Survey study	- Wide variation in funding capacity, staff size and caseloads - Little assessment, fewer treatment components in smaller than larger area programs - Unequal physician distribution - Insufficient time and training - Under-resourcing of programs leading to lack of encouragement of early referrals and prioritizing clinical care over community development work	- Easy access to vocational, educational and recreational services
Essock [34]	State Partnerships for First-Episode Psychosis Services	United States	Partnership description	- Limited public resources in behavioral health—program funding from federal government’s stimulus plan in response to recession	- Recognition by US State leaders of importance of EIP services - Sufficient capacity to train clinicians - Performance measurement - Sufficient finances to train and monitor performance
Ghio [22]	Process of implementation and development of early psychosis clinical services in Italy: a survey	Italy	Survey study	- Unequal distribution of services throughout Italy due to lack of regional and national health planning - Lack of funding - Geographic barriers to accessibility, high complexity of users’ needs and low health literacy - Implementation of specialist services within generalist model rather than specific/independent outpatient services as cheaper - Cultural resistance to change traditional treatment approaches	- None described
Gidugu [43]	Client, family, and clinician experiences of open dialogue-based services	United States	Qualitative interviews	- Younger clinicians reported lack of prior training in family therapy - Lack of resources due to fee-for-service structure which did not support multiple clinicians attending network meeting or costs of travel to clients’ homes	- Inclusive clinical network - Trained personnel available to deliver service - Organizational support in overcoming resource barriers - Support and dedicated interest from clinicians enabled delivery of intensive services
Gorrel [48]	Changes in early psychosis service provision: a file audit	Australia	Audit study	- Lack of necessary equipment such as instrument scales to enable guideline concordant care	- None described
Hardy [13]	Filling the implementation gap: a community–academic partnership approach to early intervention in psychosis	United States	Program description	- Governance issues with lack of clear decision-making processes - Sudden changes in leadership reduced outreach - Training community clinicians in evidence-based practice with rigorous fidelity requirements proved difficult for community-based social service agencies	- Reduction in high clinician caseloads - Rewarding achievement of core competencies - Mandating ongoing supervision to ensure integration of new learning - Funding streams identified to cover start-up costs - Development of state-of-art governance structure with 5 standing committees (executive, operations, evaluation, training and outreach)
Hetrick [41]	Development of an implementation guide to facilitate the roll-out of early intervention services for psychosis	Australia	Implementation guide: description of development	Lack of knowledge about model, core components, tools, and how to engage young people Lack of skill in safety and risk management Lack of clarity on professional role (e.g., responsibility to provide 24-h care, home-based care and Cognitive Behavioural Therapy (CBT)) Staff felt inexperienced and lacking confidence in delivering psychoeducation Reluctance to diagnose due to stigma, alternate beliefs about worth of model, lack of appreciation of consequences of not providing services at level needed and concern that EIP patients get 2 years of service while nonpsychotic patients get only 10 sessions Regular supervision and regular review of treatment progress not mandated Poor staff motivation due to lack of knowledge No prompts or reminders to use tools or conduct regular treatment reviews	Good knowledge of and strong commitment to engage in youth friendly practice Strong belief in EIP and possible positive outcomes of involving families and carers Clinicians aware of minimum data set requirements Desire to support and advocate for young people Desire to change service, raise awareness and bring reform
Iyer [36]	Early intervention for psychosis: a Canadian perspective	Canada	Program description	- Longer duration of untreated psychosis in patients coming from larger mental health care systems - Lack of availability of referring primary care practitioners - Services set up for 1–2 years inadequate as most functional and clinical gains made after 2 years	- Integration between research and clinical activity - Effective collaboration with referral source - Direct and rapid-response referral system
Kelly [47]	HEART—The Hounslow Early Active Recovery Team: Implementing an inclusive strength-based model of care for people with early psychosis: practice development	England	Program description	- Traditional training and clinical orientations around symptom management and adherence rather than recovery	- Collaborative partnerships with clients - Therapeutic risk taking with open-mindedness - Active elicitation of service users’ perspectives - Active involvement of local community and widespread availability of information on service - Lack of formal referral process but rather encouragement of potential referrers to contact team to discuss individual service users - Encouragement of self- and family-referral - Engagement with local young people in area - Regular team meetings to discuss recovery and engage with emerging research - Staff encouraged to be reflective in practice - Access to a ‘Right-to-work’ worker
Lester [27]	Development and implementation of early intervention services for young people with psychosis: case study	England	Mixed methods including qualitative interviews, audit of written documentation and survey	EIP perceived as elitist by CMHTs believed to poach Community Mental Health Team (CMHT) staff and having a less intensive workload due to smaller caseloads Relative deprivation and geography affect implementation Commissioners within organization saw themselves as inexperienced Team managers perceived commissioners as lacking in understanding EIP ethos Lack of collaboration with primary care trust commissioners due to poor relationships, insufficient resources and recurrent organizational restructuring and some believed primary care trust placed low priority on mental health Feeling of stigma attached to commissioners’ role which reduced potential to develop intra- and inter-organizational relationships Unable to ringfence budgets, uncertainty over funding Delayed decision-making Tension created due to staff having to meet performance targets	Consistent funding Better communication strategies between mental health teams to help different teams appreciate their relative strengths Development of exit strategies for service users at the end of 3 years with the service
Maric [28]	Implementation of early detection and intervention services for psychosis in Central and Eastern Europe: current status	Central and Eastern Europe	Survey study	- Lack of adequate infrastructure - Scarce financial support - Insufficient staff numbers - Services mostly hospital based - Lack of support from decision makers and healthcare payers - Lack of government recognition of EIP importance	- None described
McGorry and Yung (29)	Early intervention in psychosis: an overdue reform	Australia	Descriptive	- Lack of resources for specialist mental health services - Targeting on chronic schizophrenia means broad diagnostic range of patients cannot access specialist services, particularly diagnostic ambiguity of early psychosis - Lack of policy support at State and Federal level - Belief at bureaucratic level that proven EPPIC model cannot be replicated - Lack of integration of specialist mental health with primary care and other community agencies	- EPPIC program and National Early Psychosis Project created strong foundation for systematic reform in Australia - Three national and 3 international Early Psychosis Conferences providing strong networks to support growth of research and service reform
North [32]	Design, implementation, and assessment of a public comprehensive specialty care program for early psychosis	United States	Mixed methods including survey and audit of records	- Core clinical staff not in place within first 5 months of program - High staff turnover delayed establishment of clinical teams - Difficulties in patient recruitment and retention threatened funding, taking 1 year to reach patient capacity - Restrictive insurance and income eligibility criteria allowed only 1 of 100 patients to enroll who would otherwise be eligible - Extensive staff efforts to engage and retain patients diverted time from clinical provision - Restrictive exclusion criteria resulted in impoverished sample with lack of access to transport, further limiting ability to engage	- None described
Pinfold [24]	Audit of early intervention in psychosis service development in England in 2005	England	Audit study	-Lack of out-of-hours support, designated acute beds and input from child mental health services - Few teams resourced to provide comprehensive service - Half of teams funded only to case manage with no early detection capacity - Few teams measured DUP - Inconsistencies between teams in standardized measurement - One-fourth of teams deviate from specialist service model but did not formally apply for service flexibility	- National research programs aid understanding of impact of EI on patients and families
Powell [31]	Implementing coordinated specialty care for first episode psychosis: a review of barriers and solutions	United States	Literature review	- Variation in programs creates problems with evaluation and program fidelity - High staff turnover - Working in EIP, staff vulnerable to feelings of worry, anxiety and frustration and job-related stress linked to financial concerns - Implementation difficult in rural communities with isolation, fewer resources, inadequate number of staff, self-sufficiency and lack of trust of outsiders - Allocation & retention of resources problematic - Lack of centralized source of funding for EIP - Variability in financial support at State level	- Time and financial investment in staff training - Prevent turnover through emotional support for supervisors, supportive leadership, improved workplace environment, adequate initial training and ensuring staff have realistic view of job prior to starting
Reilly [37]	Implementation of a First presentation psychosis clinical pathway in an area mental health service: the trials of a continuing quality improvement process	Australia	Program description	- Inadequate documented evidence base for guidelines and pathway - Initial failure to establish measurable objectives and ensure active management involvement - Difficulty in engaging psychiatrists and acute care clinicians in practice change - Over-ambitious expectations of clinical practice - Excessive and duplicative documentation - Lack of specificity in training, targeting and duration of training - Lack of additional support to complete formal evaluation in routine clinical care - Inability to evaluate service improvement at patient outcome level - Disruption of process due to key staff and external circumstances	- None described
Tiffin and Glover (23)	From commitment to reality: early intervention in psychosis services in England	England	Mixed methods including qualitative interviews and examination of routine records	- Complexity in process of commissioning of services - Funding deficits within NHS health Trusts due to increased staff costs resulting in funding for EIP in some areas being frozen or reduced - Low caseloads and referrals due to original overestimates of case prevalence in nonurban areas - Limitations in capacity	- Effective leadership - Commitment of service commissioners to EIP - Research and governance activities - Regional EIP “champions” - At least one area where EIP services were developed early, providing “geographic epicenter” for developments
White [49]	Essential components of early intervention programs for psychosis: Available intervention services in the United States	United States	Qualitative interviews	- Geographic distribution skewed toward U.S. west coast driven by shift in funding in California with 20% of all funds from specific tax spent on prevention and EI in mental health - Under-utilized EIP components include outreach services and communication protocols with inpatient units	- None described

Abbreviations: EIP; early intervention in psychosis.

#### Countries of origin

Most studies were based in high-income countries, including the United States (*n* = 6), England (*n* = 5), Australia (*n* = 4), Canada (*n* = 3), Italy (*n* = 2), and Switzerland (*n* = 1). One originated in Central and Eastern Europe (*n* = 1), while one included descriptive information from services across the world.

#### Methods

A variety of methodologies were employed, including descriptions of EIP implementation (*n* = 8), qualitative (*n* = 3), survey (*n* = 3), mixed methods studies (*n* = 3), narrative reviews (*n* = 2), audits (*n* = 2), a case study and one feasibility study. See Table 2 for a full list of study methodologies.

#### Participants and study sites

Studies employed a variety of participant groups to assess views and experiences of implementation. In 11, there was no direct sampling of any participant group. Instead, the papers comprised authors’ own descriptions or reviews of service or program implementations. In three, patients were directly sampled and in two, participants were EIP clinicians. In the remaining six, participants were described as representatives of services, EIP professional experts, senior EIP program decision makers, program leads, a mixed sample of patients, families, and clinicians, or there was no description of participant type.

In six studies, there was no sampling of specific EIP sites. Of the remaining 17, the mean number of sites sampled was 31 (range: 1–152), with 8 sampling only 1 site. Ghio et al. surveyed 152 mental health centers in Italy, Tiffin et al. sampled 118 teams supported by 53 National Health Service (NHS) Trusts, and Pinfold et al. sampled 117 EIP teams using a self-report audit tool in eight English regional development centers.

#### EIP services

A variety of service models were included in studies. In 12, the authors presented macro-level details from either a variety of EIP services across international countries, across countries within a region, or across regions within a country. In the remaining 11, information on individual services and their components was available (Table 3). These studies include a variety of service models, including hub and spoke models [28,37], standalone teams [27,30–32,44], or services that focused on collaborative partnerships [14,40,50]. Each offered early intervention, a range of psychosocial services, psychiatric and medication reviews and often some form of assertive case management.

**Table 3.** Information on individual EIP services and their key components.

Author	EIP service	Key service components
Baumann [26]	Treatment and Early Intervention in Psychosis (TIPP-Lausanne)	Staffing requirements (case manager (3.5 Full Time Equivalent (FTE)), consultant psychiatrists (0.5 FTE), intern psychiatrists (0.6 FTE) and psychologists (0.6 FTE). Treatment provided to patients aged 18–35 with no previous treatment with antipsychotic medication for >24 weeks, and crossed psychosis threshold to CAARMS criteria Case-management model contact with patients within 48 h. Case managers have limited caseload (max 30 patients) and are trained in assertive case management Multidisciplinary treatment offered involving psychiatrist, social workers and psychologists Two home visits a week offered in cases of crisis Additional Assertive Community Team provide: (a) assessment and engagement of patients who are treatment refractory; (b) transitory treatment when close monitoring is needed at frequency exceeding twice per week; and (c) alternative to hospital admission when relapse occurs Available interventions: psychoeducation, psychological intervention for cannabis abuse, multi-familial sessions, prospective monitoring of medication side-effects, cognitive assessment and remediation, supported employment, case management manual Outcomes monitored through prospective data collection
Brabban [42]	Northumberland EIP service	Hub and spoke model Single, central clinical psychologist supporting five care coordinators (spoke nurses) Workers recruited from existing CMHTs in locality and remained housed within these teams No evaluation of adherence to medication Psychiatrist appointed 2.5 years after service became operational so little influence over prescribing or medical involvement in this period Team lacked support worker Five care coordinators—three had qualifications in psychosocial interventions for psychosis and one was undergoing training Caseloads capped to allow implementation of individualized, formulation-driven approach Regular supervision from clinical psychologist
Cocchi [25]	Five EIP centers in Departments of Mental Health of Milan, Rome, Grosseto, Salerno, and Catanzaro	Milan site acted as coordinating center for other four sites Staff recruitment: no less than three staff (including at least one psychiatrist and one psychologist) One meeting/month with potential sources of referrals (GPs, emergency services, pediatricians, and child neuropsychiatrists) Patients aged between 17–30 years after first contact with any public mental health service within catchment area for FEP, with DUP < 24 months. Service also accepted UHR patients. Affective psychosis was an exclusion criteria Treatment duration 3 years. Comprehensive, tailored and flexible interventions—community-based case management, individual psychoeducation and motivational sessions, CBT, family support, therapeutic group activities (anxiety management, substance abuse prevention), social groups (e.g. music, multimedia, computer training) and supportive interventions on employment, school, compliance with medication and planning of recreational activities
Essock [34]	RAISE Partnership	Staffing requirements (full-time team leader and supported employment-education specialist, recovery coach [0.5 FTE] and psychiatrist [0.2 FTE]) Anyone in area meeting the eligibility criteria offered services, regardless of insurance status Team not responsible for filling its own caseload; separate outreach and referral staff responsible for outreach and eligibility Funding for recurring costs and implementation costs (e.g., acquiring and furnishing space, training and staffing) Services delivered include traditional psychiatric services, support services (e.g., employment and education), and clinical case management Weekly team meetings Teams embedded within existing mental health programs Local agency leadership responsible for supervision and regulatory oversight
Gidugu [43]	“Collaborative Pathway”	Emphasizes rapid and early intervention Adapting treatment to meet changing and specific needs of individuals Providing psychotherapeutic treatment for all patients within personal support systems Seven principles:( (a) Provision of immediate help; (b) social network perspective; (c) flexibility and mobility; (d) responsibility; (e) psychological continuity; (f) tolerance of uncertainty; and (g) dialogism
Hardy [13]	Prevention and Recovery from Early Psychosis (PREP)	2-year service for patients aged 12–35 after recent onset psychosis or those at ultra-high risk of psychosis Began from academic-community partnership and advocacy organization partnership which provide community outreach and education Recovery-based service: diagnostic intake and assessment; collaboration with family and patients to triage services; algorithm-based medication management; strength-based care management, individual CBT; psychoeducational multi-family groups, vocational/educational support and substance use treatment Outcomes monitored
Hetrick [41]	The Early Psychosis Prevention and Intervention Center (EPPIC)	24-h access via dedicated mobile early detection and home treatment team with timely assessment for FEP patients Minimum 2-year tenure of service, with option of care extending to 5-years post-entry and access to youth-specific inpatient unit Psychoeducation and support provided for patient and family on an initial, continuing and ‘as needed’ basis through individual work, group programs and family participation groups Treatment response and adherence reviewed regularly. Patients seen weekly by case manager and fortnightly by doctor in the early recovery phase Case managers (caseload of 15–20 each) provides access to a range of evidence-based psychological therapies dependent on need (e.g., CBT, Cognitively Orientated Psychotherapy in Early Psychosis, Cannabis and Psychosis) Family work provided on regular basis including psychoeducation, regular family meetings relevant to phase of illness
Iyer [36]	Prevention and Early Intervention Program for Psychosis (PEPP)	Targets FEP patients aged 14–35 years with affective or nonaffective psychosis who have had no more than 1 month’s previous antipsychotic treatment, without organic brain damage, a pervasive developmental disorder, an IQ below 70 or epilepsy and do not have substance-induced psychosis. Comorbid diagnosis of substance abuse not an exclusion criterion. Outreach program to educational professionals Quick response protocol and open referral system (patients and families can self-refer) with no forms required and referral response within 72 h. Trained intake clinician responds to all referrals and conducts initial evaluation. Within a week a psychiatrist conducts a full assessment to establish diagnosis and initiate or adjust pharmacological treatment. Phase-specific, specialized, developmentally informed comprehensive treatment provided for first 2 years after diagnosis Treatment includes intensive case management who maintain regular contact (twice per week in first 2 months, no less than once/month at any point in follow-up). Case managers provide psychoeducation and supportive therapy. Caseloads are 20–25 patients Other treatments include medication management, physical health interventions, PEPP housing project, PEPP Family Psychoeducation Program, multiple-family group treatment, family support groups, psychosocial interventions (CBT, Individual Placement and Support, art/dram expression sessions, “Recovery through Activity and Participation,” “Group CBT for Social Anxiety,” “Youth Education and Support,” and “Work Preparation Group”) PEPP assessment protocol aims to integrate clinical and research/assessment activities and conducts a Sharing Knowledge Day where research findings are discussed and shared with patients and families.
Kelly [47]	Hounslow Early Active and Recovery Team (HEART)	Targets people aged 14–35 who develop psychosis, providing treatment for first 3 years of contact with mental health services Staff members include: one team manager (0.5 Whole Time Equivalent (WTE)), one CBT-trained senior nurse, three community mental health nurses, one occupational therapist, one social worker, five sessions/week from consultant psychiatrist, eight sessions/week from middle-grade psychiatrist in training, six sessions/week from clinical psychologist, one support, time, and recovery worker, one community support worker, two sessions/week from a “Right-to-Work” worker, four sessions/week from a community development worker for black and ethnic minority community members Team have access to premises of local youth counseling service on weekly basis Interventions provided: psychosocial interventions (e.g., structured relapse prevention), medication management, cognitive behavioral interventions, relaxation and anxiety management skills, activities of daily living assessments and goal planning, coping strategy work plans, family interventions.
North [32]	The Enhanced Program for Early Psychosis (ePEP)	Service provided to patients with referring clinical diagnosis of primary psychosis with ≥1 psychotic symptom during the current episode, aged 15–30, first onset of psychosis began within last 2 years and duration of psychotic symptoms >1 week, ineligible for Medicaid/Medicare and < 200% of federal poverty level status, and living within commuting distance and anticipated availability to attend the clinic for ≥12 months Two clinical provider teams, each serving 30 patients with 4 staff (full-time team leader, full-time case manager/healthcare coach, full-time individualized placement and support specialist & half-time family peer support specialist) Case manager provides intensive outreach, recruitment and retention services Placement and support specialist provides vocational and educational rehabilitation services Family peer support specialist serves as a recovery coach, assisting patients with attending appointments, procuring medication and managing daily activities
Reilly [37]	The First Presentation Psychosis Clinical Pathway	Initial completion of the Crisis Triage Rating Scale Daily contact for the first 4 weeks with completion of symptom measures Review by a consultant psychiatrist and completion of a family/carer interview within 1 week Structured medication decision points and formal clinical review with consultant psychiatrist at 3-weekly intervals until 12 weeks Allocation of a case manager for all patients presenting with FEP

Abbreviations: EIP; early intervention in psychosis; FEP, first episode psychosis.

### Narrative synthesis

Narrative synthesis identified three domains: (a) system; (b) service; and (c) staff, with 14 associated subdomains. Domains and subdomains are described in Table 4, and Table 2 outlines each barrier and facilitator identified in each study.

**Table 4.** Identified barriers and facilitators of EIP service implementation.

Domain	Subdomain	Facilitators or barriers	Number of studies citing subdomain
System	Funding	Ringfencing of budgets	14
		Centralized funding source	
		Complexity in commissioning services	
		Funding and resource deficits, uncertainty, and inconsistency	
		EIP funding threatened by deficits in other areas of health system	
		Sufficient funding of EIP start-up costs	
		Funding programs for sufficient lengths of time (e.g., 1–2 years only)	
		Regional variability in funding within countries	
	Preimplementation services and structures	Strength of existing healthcare system and individuals’ to access to services	11
		Unequal distribution of services within country	
		Health inequalities and impoverished patient groups within EIP areas	
		Geographic barriers	
		Ability to adapt to variations local conditions and constraints	
	Organizational support and structures	Top-down support and willingness to overcome resource barriers	7
		Efficient governance structures with abilities to enact quick decisions	
		Effectiveness of leadership and development of intra- and inter-org relationships	
		Understanding and commitment to EIP ethos	
		Recurrent changes in leadership and organizational restructuring	
	Political interest	Lack of recognition of EIP value or interest in EIP	4
		Lack of policy support	
Service	Collaboration and communication with outside groups and services	Academic partnerships and integration of academic and clinical activity	13
		Clinical networks	
		Partnerships with local and provincial providers	
		Communication with non-EIP professionals	
		Communication and collaboration with service users and families	
		Availability of information on EIP service in communities	
	Coherence of EIP program	Strength of definition of EIP program	9
		EIP program variations leading to difficulties in evaluation of fidelity	
		Existence and adequacy of provisional implementation guidelines	
		Clarity of professional roles and definitions	
		Mandating of regular review of treatment progress	
		Suite of available and accessible assessments and treatments	
		Over-ambition in clinical practice expectations	
		Ability to record and acknowledge deviations in implementation guidelines	
	Assessment and measurement	Lack of available measures and instrument scales	9
		Sufficient financing to train and monitor performance monitoring	
		Measurement of DUP	
		Inconsistent measurement of performance and outcomes between EIP teams	
		Elicitation of service users’ perspectives	
		Excessive and duplicative documentation requirements	
		Assessment of fidelity to treatment and training for clinicians to enact	
		Staff incentives and rewarding achievement of core competencies	
	Training capacity	Specificity, capacity, targeting, and duration of available training	8
		Traditional training focuses on management and adherence instead of recovery	
		Training availability in evidence-based practices	
	Caseloads	Capping of caseloads	6
		Staff size	
		Availability of out-of-hours service	
		Time spent engaging service users diverted from clinical work	
	Referral and discharge	Availability, linkages and communication with GPs and other referrers	6
		Encouragement of referrers to directly discuss service users with EIP team	
		Rapidity, directness and ease of referral	
		Possibility of self- and family referral	
		Referral eligibility criteria (e.g., broad vs. restrictive)	
		Exit strategies for patients	
		Recruitment and retention tied to service funding	
	Staff supervision	Regularity of supervision	5
		Mandating of supervision	
		Regular team meetings	
	Infrastructure	Virtual locations	2
		Lack of physical sites	
		Poor adaptation of facilities to needs of young people	
		Infrastructure availability	
Staff	Staff attributes	Competence, motivation, confidence, and experience	9
		Reluctance to diagnose due to fear of stigma, or lack of knowledge	
		Ability to acquire and enact EIP philosophies	
		Willingness to advocate, raise awareness for and reform EIP services	
		Concern that non-EIP patients suffer as resources diverted to EIP teams	
		Support for, knowledge of, commitment, and dedicated interest in EIP	
		Skill in safety and risk management	
		Awareness of minimum data set requirements	
		Collaborative and engagement efforts with patients and families	
	Recruitment and retention	Staff turnover due to financial concerns, stress, anxiety, and worry	6
		Sudden changes in team leadership	
		Speed of staff recruitment and appointment	

Abbreviations: EIP; early intervention in psychosis.

#### System barriers and facilitators

##### Funding

The most commonly cited barrier was insufficient funding (cited in 14 studies [11,27,33,37,42,43]). Under-resourcing of programs led to insufficient time and scope for staff training, prioritization of clinical work over community development and outreach [38], insufficient staffing [43], and financial concerns amongst staff [34]. Program funding models within countries varied [34,38]. In the United States, private insurer models existed rather than centralized financing [34]. This threatened service sustainability as insurers required the demonstration of treatment indication, reimbursing direct clinical care only, and requiring programs to operate without financial loss [34].

Services were often guaranteed future spending dependent upon achieving specific outcomes. In the United States and England, funding continuation depended on the number of engaged patients [44] or the meeting of caseload targets [42]. Teams often struggled to recruit and retain patients in their first year and staff responded by restricting age eligibility criteria, discharging patients early, or imposing waiting lists [42].

Complex service commissioning systems were reported in England, where a single EIP team negotiated with numerous Primary Care Trusts [46]. EIP commissioners reported recurrent organizational restructuring as an impediment to partnership-building across health and social care sectors, mental health as a low priority, and an inability to ring-fence mental health budgets [42].

##### Preimplementation services and structures

The strength and availability of existing services affect the ease with which new models can be established. In Italy, EIP diffusion was only 20–30% [39], where implementation heterogeneity was a consequence of chronic regional under-investment and local deprivation. Services in regions of high deprivation face greater challenges due to complex housing needs, high unemployment, higher psychosis incidence [51], harder-to-reach groups like refugees and asylum seekers, and have fewer opportunities to involve voluntary and community services [42]. Rural isolation and inaccessibility will likely lead to unequal physician distribution [38].

Low-income countries face the greatest implementation problems, in particular due to a greater historical reliance on institutionalization [11]. In Central and Eastern Europe, mental health expenditure ranged from 1.4 to 8%, and the number of psychiatrists ranged from 1.3 to 13 per 100,000 population [43]. In Germany, mental health expenditure is 11% and there are 15 psychiatrists per 100,000 population.

##### Organizational support and structures

Effective leadership and good governance structures facilitate implementation. Regional English EIP ‘champions’ (i.e., teams who developed early) helped guide and instil optimism in under-developed teams [46]. Speedy decision-making and belief in an EIP ethos were regarded as necessary components within governance structures [42]. A program in San Francisco established executive, operations, evaluation, training, and outreach standing committees, giving each their own charter, scope of competency, membership, chair, meeting schedule and performance metrics. This adoption of an established business model within EIP governance structures could improve service efficacy [14].

##### Political interest

Political disinterest stymies EIP development. The emergence of strong evidence on EIP effectiveness from the OPUS trial convinced politicians to financially support EIP programs in Denmark [11], but health departments in Bosnia and Herzegovina and the Ukraine published mental health strategies referencing EIP, without timeframe commitments [43]. U.S. state leaders in Maryland and New York recognized research that established the feasibility of EIP teams, a recognition accompanied by funding and promises to expand services within both states [50]. Political recognition is vital at all levels of government.

#### Service barriers and facilitators

##### Collaboration and communication with outside groups and services

Thirteen studies discussed the need for effective collaboration and communication links with other organizations. National and international conferences can foster clinical and academic networks [33]. Clinical academics can provide data to help establish programs and outcome monitor [36], while services improve study recruitment [27]. Montreal’s PEPP program used research assessments to help set treatment goals and research was shared with patients [31]. Pinfold et al. [45] caution however that research is not a substitute for overcoming structural problems like inequitable access and service incapacity and Cheng et al. [36] reported that provincial Ontario EIP advocacy networks were more influential in service initiation than direction from research.

The importance of service outreach was common across studies with linkages described with schools, employment agencies, child and adolescent psychiatry, drug and alcohol services, primary care, child and youth mental health agencies, youth shelters, housing services, fundraising officials and marketing firms [11,33,36]. Collaborations can increase referrals, improve access to hard-to-reach patients, and raised patient and family satisfaction [36]. Small teams particularly benefit from such partnerships [38], and allowed patients who did not meet an EIP team’s inclusion criteria to receive appropriate community referrals [31].

##### Coherence of the EIP program

The coherency of the EIP model matters. These models should draw from existing evidence, incorporate workers’ vision and measure fidelity [11]. While fidelity measurement of fidelity is important, tension was noted between creating services derived from gold standards versus adaptations to local contexts. Cheng et al. [36] reported team leaders’ frustration at a lack of area-specific guidelines, but where local guidelines existed, some clinicians found these too restrictive. Given the clinical and biological variability of psychotic disorders and the likelihood that the course and outcome is affected by regional differences, regional guidelines could prove clinically effective. In an audit of 117 EIP teams in England [45], a quarter of teams deviated from the policy implementation guide, but few formally applied for fidelity flexibilities. EIP models can adapt to specific contexts if there is concurrent implementation evaluation [11], and if patient outcomes and cost-effectiveness remain equivalent. Nonetheless, Pinfold et al. [45] caution that large deviations within an environment of funding deficits and access inequity may lead to insufficient resourcing, affecting the ability to provide the comprehensive services and eroding the integrity of the original EIP model.

##### Assessment and measurement

Prospective patient outcome, treatment fidelity and service performance monitoring are important facilitators. Consistency in standardized patient outcomes strengthens the ability to compile evidence on value. Demonstrations of service and treatment fidelity improve future replication efforts.

Measurement issues were described in two studies. Inadequate data collection in Italian EIP teams was linked to a lack of completed or available standardized assessments [37]. An audit of English services found few teams measured DUP and there were inconsistencies in standardized measures [45]. Standardized DUP scales include a checklist [52] measuring the emergence of first noticeable symptoms, psychosis and treatment-seeking. The Interview for the Retrospective Assessment of the Onset of Schizophrenia [53] assesses symptoms and impairment at the onset of emerging psychosis and the Nottingham Onset Schedule [54] defines onset as the time between first reported change in mental state and the development of psychotic symptoms. It allows measurement of treatment delays, duration of untreated illness, and duration of both untreated emergent and untreated manifest psychosis. DUP measurement is varied and complex, for example, DUP end can be defined differently, for instance, the point of antipsychotic prescription, referral to nonpharmacological treatment or if there are affective components, when antidepressants are given. A recognition that inconsistent measurement impedes service continuation, and that conversely, excessive and duplicative documentation burden staff, led an Australian service to develop a clinical pathway integrating outcome measurement into routine documentation procedures [35]. The San Francisco PREP program trained staff in rigorous data collection, developing an electronic health record that enabled data sharing within teams and with community partners [14].

Service fidelity measurement is expensive but possible using routinely collected, service data [29,48], where structural (e.g., staffing) and care processes (e.g., presence of completed side-effect checklists) can be assessed [49]. Staff reminder prompts improve completion and entry of outcome measures [30]. Most importantly, a recognition of the value of patient and service monitoring is required, alongside the provision of funding and training [50].

##### Training capacity

Training in psychosocial interventions was a facilitator in eight studies. Teams can suffer from high staff turnover [34] and top-up training can improve retention. In Italy, no specialized training was provided in 26% of 152 teams surveyed [39], while in Northumberland [28], most EIP care coordinators were trained in psychosocial interventions, a fact the authors argue as central to the team’s success.

The opportunity to receive training can attract new staff and a diversity of training perspectives provide more tools with which to assist recovery [40]. Training costs should be factored into start-up and expansion financing, but teams can develop partnerships to help cover training costs, for example the US RAISE program collaborated with academics to train staff [50].

##### Caseloads

Small caseloads are recommended as the gold standard in EIP delivery. UK EIP guidelines [55] recommend 15 patients per care coordinator. Ontario’s implementation policy did not specify a caseload target and as a result, 25% of programs reported staff caseloads greater than 25 [38]. Higher caseloads can lead to delays in intervention delivery [28], and limitations on outreach activities which serve to increase referrals [42]. High caseloads are not universal, however. Of the 118 teams in operation in England in 2006, only one region was served by teams carrying caseloads approaching the target. This was due to an overestimation of rural prevalence, limits on teams’ capacity, and lower-than-expected referral rates [46]. Prevalence estimates are a common planning requirement [56], but Tiffin et al. caution that caseloads are unlikely to be established until teams are in operation for 3 years [46]. A perception that EIP teams carry small caseloads caused tensions with generic community mental health teams who viewed EIP staff as carrying less intensive workloads. Communication between teams ensure staff appreciate each other’s relative strengths [42].

##### Referral and discharge

The strength of teams’ referral links improves patients’ rapid access to services. Patients often first have contact with mental health services at crisis point, or may be initially referred to CMHTs, potentially resulting in increased DUP [57]. Referrals from large mental health systems are associated with longer DUP, where care pathways may be more complicated [31]. Periodical meetings with potential referrers can be effective [37]. Links with local emergency departments prevented hospitalizations and minimized the potentially traumatic effects of encountering care within emergency or inpatient settings [31]. Family and self-referral improved service uptake and decreased help-seeking delay [31,37]. Reductions in documentation, along with the appointment of trained intake clinicians later involved in treatment, improved access and helped establish engagement [31,32].

##### Staff supervision

Several issues arose regarding supervision of staff. In the Italian system, the provision of clinical supervision was low, with at most 12.5% of teams offering supervision in northern regions, with no provision in the south. The Australian EPPIC and San Francisco PREP models noted that nonmandating clinical supervision impeded implementation [14,30]. EPPIC developed a workforce development plan requiring clinical supervision on a minimum fortnightly basis. In a Northumberland EIP service [28], a clinical psychologist provided supervision to all practitioners, regardless of specialism. Regular supervision for staff in rural regions reduces feelings of isolation [34], while hub and spoke models led to some confusion as to who was responsible for the supervision of spoke staff embedded within CMHTs [36].

##### Infrastructure

In Central and Eastern Europe, the most commonly cited limitation to implementation was inadequate infrastructure, with many services operating within hospital settings [43]. Staff may lack appropriate facilities within hub and spoke models specifically due to the often virtual operation of hubs. In Northumberland [28], this impeded attempts to improve patient engagement. As the average EIP patient is often younger than in general mental health services, youth-friendly, low stigma out-patient settings that are easily accessible are vital.

#### Staff barriers and facilitators

##### Staff attributes

The skills and competencies required to work within EIP services was described in nine studies. A range of competencies were described: safety and risk management; EIP model knowledge; treatment knowledge; ability to engage with young people; confidence in treatment delivery [28]; understanding recovery principles [32]; ability to instil therapeutic optimism; willingness to diagnose; belief in EIP ethos; enacting service change [30]; creativity in patient engagement [27]; and ability to adapt therapies; [40]. EIP services often adopt collaborative approaches, focusing on goals, resources and achievements, often a departure from traditional approaches [32]. Similarly, staff can be expected to share therapeutic roles and responsibilities, regardless of professional background [42]. This necessitates a willingness to engage in “mundane” tasks, like visiting cinemas or offering lifts.

##### Recruitment and retention

Issues relating to staff recruitment and retention were discussed in five studies. Powell et al. [34] reported that recruitment was a major barrier to implementation in the United States, suggesting teams identify the skills that best complement existing team dynamics prior to role-filling. High staff turnover was seen as a concern, particularly in the early months of teams’ existence [44], leading to disruptions in the establishment of team processes [35]. Due to under-financing, staff may work beyond capacity for long periods, feel excessive pressure to meet targets, leading to the erosion of morale and good will [42]. Emotional support, supportive leadership styles and positive work climates are necessary [34].

## Discussion

### Main findings

This systematic review and narrative synthesis provides data from 23 studies and identified three over-arching domains that influence implementation: system, service, and staff-level factors, with 14 associated subdomains. There was considerable overlap between subdomains. Barriers and facilitators to implementation were common across many countries and regions.

The most common barrier was funding. This issue played a demonstratable role in many subdomains, for example, the preimplementation landscape, staff training, referral and outreach practices, caseload targets, and the ability to monitor and evaluate services. Funding deficits are likely partly fueled by political disinterest. It is of concern that insufficient budgets, and the indexing of budgets to performance, caused service adaptations without associated evaluation, threats to fidelity and the ability to replicate and “scale-up” services. That budgeting issues underlie so many subdomains also suggests that sufficient funding of EIP services, while not the only antidote, could assist in overcoming many attendant barriers. Funding programs in a post-COVID context may become difficult, particularly in low- and middle-income countries (LMICs). In LMICs EIP prioritization may be achieved via increased political will, legislative change, better allocation of resources, and organization, increasing the mental health workforce, reducing funding to hospitals while increasing community spending, and greater patient involvement in service design. In Western countries, the same principles apply, coupled with progressive taxation based on income and wealth, and legislating for minimum mental health budgets of at least 15%.

It is a truism that the existing healthcare landscape will significantly influence implementation. The extent of existing staffing levels, governance structures, clinical networks, collaborations, evaluative capacity, and prior investment predict success. The studies in this review did not describe “treatment as usual” prior to EIP implementation, but the barriers identified align with common macro-level barriers in health service reform more generally, such as inertia to change, regulatory challenges, operational complexity, and unclear financial and governance processes [58,59]. The symbiotic relationship between and within domains suggests pre-implementation evaluation, using validated measurement, of all aspects of the health system context could aid the development of EIP models [60], and better predict the local adaptations necessary.

The system domains are generic to most health and mental health services. Lessons could be drawn from other specialities. Stroke care also emphasizes early intervention and underwent substantial reform in London in 2008 [61]. Program leaders wielded significant political power, required rigorous performance measurement to achieve accreditation, and previous system failures led to a focus on implementing small numbers of only essential priorities [62]. While lessons from other medical specialities necessitating early intervention are useful, EIP barriers and facilitators could also prove useful in other settings. Nonetheless, services for severe mental illnesses face a unique set of challenges [16].

Many of the service and staff-level domains were specific to EIP provision, such as the necessitation of strong referral partnerships and collaboration across and between governmental organizations. EIP models require clear supervisory lines of support and training so staff embedded in CMHTs and rural areas do not face isolation. The studies included in this review did not provide specific information on staff training, however such programs exist (e.g., the NHS’s Health Education England elearning course and the OnTrack New York initiative). A future review on their specific components and differences could be useful. The literature highlights a set of unique EIP competencies with staff required to embrace new ways of working, and a fluidity in professional identity. Finally, funding systems must recognize the developmental and outreach aspects of EIP services, alongside the attainment of clinical targets [42]. Some guarantees of consistent financial support could reduce uncertainty and build trust amongst staff.

### Limitations

There are several limitations. We included studies available in English only, likely underrepresenting non-English speaking countries. We included implementation descriptions with no associated methodologies, which precluded quality assessment of all studies. Of studies employing methodologies, much detail on barriers and facilitators was extracted from discussions, although this real-world information is likely a good representation of experience. This review describes findings from healthcare systems where EIP services are implemented which will differ to those where no EIP exists. Few studies included formal assessments of implementation, utilized implementation frameworks or applied fidelity measures, limiting the strength of evidence, and there was wide variability in the type of EIP descriptions published. A strength however is that many of the domains identified in the narrative synthesis mirror items in Addington’s [8] fidelity scale, suggesting ecological validity. We adopted a bottom-up approach to data analysis, guided by thematic analysis, rather than adopting an implementation framework like the Consolidated Framework for Implementation Research to guide data analysis. Such an approach may have yielded differing results. Finally, stakeholders’ views on EIP efficacy did not emerge as a theme within survey of the literature, but a belief in a lack of EIP efficacy could lead to views that EIP investment is unjustified. Unfortunately, we cannot comment on this.

## Conclusion

Despite these limitations, this review highlights the generic and specific challenges to EIP implementation and sustainability with practical implications. The commonalities between domains suggest multiple potential avenues through which implementation can be driven. EIP has promoted recovery and increased access to care, but coverage is inconsistent. A better understanding of the EIP implementation gap and the ways in which it can be overcome, helps ensure these services can be accessed by a wider range of patients and families.

## Data Availability

Data that supports the findings of this study are available from the corresponding author upon reasonable request.

## Supplementary Material

To view supplementary material for this article, please visit http://doi.org/10.1192/j.eurpsy.2021.2260. click here to view supplementary material

## References

[1] Nielsen RE, Uggerby AS, Jensen SO, McGrath JJ. Increasing mortality gap for patients diagnosed with schizophrenia over the last three decades—a Danish nationwide study from 1980 to 2010. Schizophr Res. 2013;146(1–3):22–7. 23523021 10.1016/j.schres.2013.02.025

[2] Salomon JA, Vos T, Hogan DR, Gagnon M, Naghavi M, Mokdad A, et al. Common values in assessing health outcomes from disease and injury: disability weights measurement study for the Global Burden of Disease Study 2010. Lancet. 2012;380(9859):2129–43. 23245605 10.1016/S0140-6736(12)61680-8 PMC10782811

[3] Jongsma HE, Gayer-Anderson C, Lasalvia A, Quattrone D, Mulè A, Szöke A, et al. Treated incidence of psychotic disorders in the multinational EU-GEI study. JAMA Psychiatry. 2018;75(1):36–46. 29214289 10.1001/jamapsychiatry.2017.3554 PMC5833538

[4] Jääskeläinen E, Juola P, Hirvonen N, McGrath JJ, Saha S, Isohanni M, et al. A systematic review and meta-analysis of recovery in schizophrenia. Schizophr Bull. 2013;39(6):1296–306. 23172003 10.1093/schbul/sbs130 PMC3796077

[5] Marshall M, Lewis S, Lockwood A, Drake R, Jones P, Croudace T. Association between duration of untreated psychosis and outcome in cohorts of first-episode patients: a systematic review. Arch Gen Psychiatry. 2005;62(9):975–83. 16143729 10.1001/archpsyc.62.9.975

[6] Larsen TK, Melle I, Auestad B, Haahr U, Joa I, Johannessen JO, et al. Early detection of psychosis: positive effects on 5-year outcome. Psychol Med. 2011;41(7):1461–9. 20942996 10.1017/S0033291710002023

[7] Demyttenaere K, Bruffaerts R, Posada-Villa J, Gasquet I, Kovess V, Lepine JP, et al. Prevalence, severity, and unmet need for treatment of mental disorders in the World Health Organization World Mental Health Surveys. JAMA. 2004;291(21):2581–90. 15173149 10.1001/jama.291.21.2581

[8] Addington D, Norman, R., Bond, G.R., Sale, T., Melton, R., McKenzie, E., Wang, J. Development and Testing of the First-Episode Psychosis Services Fidelity Scale. Psychiatr Serv. 2016;67(9):1023–5. 27032665 10.1176/appi.ps.201500398

[9] Maric NP, Petrovic SA, Raballo A, Rojnic-Kuzman M, Klosterkötter J, Riecher-Rössler A. Survey of the European Psychiatric Association on the European status and perspectives in early detection and intervention in at-risk mental state and first-episode psychosis. Early Interv Psychiatry. 2019;13(4):853–8. 29882272 10.1111/eip.12682

[10] Addington DE. Performance measures for evaluation and quality improvement in the care of individuals with a First Episode Psychosis. Rockille, MD: Agency for Healtcare Research and Quality; 2011.

[11] Csillag C, Nordentoft M, Mizuno M, McDaid D, Arango C, Smith J, et al. Early intervention services in psychosis: from clinical intervention to health system implementation. Early Interv Psychia. 2018;12:757–64. 10.1111/eip.12514 29143456

[12] McGorry PD. Plenary session: the next stage for early intervention: transdiagnostic, personalised, universal. Early Interv Psychia. 2016;10(S1):3–6.

[13] McGorry PD, Ratheesh A, O’Donoghue B. Early intervention—an implementation challenge for 21st century mental health care. JAMA Psychiatry. 2018;75(6):545–6. 29801060 10.1001/jamapsychiatry.2018.0621

[14] Hardy KV, Moore M, Rose D, Bennett R, Jackson-Lane C, Gause M, et al. Filling the implementation gap: a community-academic partnership approach to early intervention in psychosis. Early Interv Psychia. 2011;5(4):366–74. 10.1111/j.1751-7893.2011.00310.x PMC3205471 22032550

[15] Pelosi AJ, Birchwood M. Is early intervention for psychosis a waste of valuable resources? Br J Psychiatry. 2003;182:196–8. 12611780 10.1192/bjp.182.3.196

[16] Millard C, Wessely S. Parity of esteem between mental and physical health. BMJ. 2014;349:g6821. 25398394 10.1136/bmj.g6821

[17] Eccles MP, Mittman BS. Welcome to Implementation Science. Implement Sci. 2006;1(1):1.

[18] Best A, Greenhalgh T, Lewis S, Saul JE, Carroll S, Bitz J. Large-system transformation in health care: a realist review. Milbank Quarter. 2012;90(3):421–56. 10.1111/j.1468-0009.2012.00670.x PMC3479379 22985277

[19] Paul CL, Ryan A, Rose S, Attia JR, Kerr E, Koller C, et al. How can we improve stroke thrombolysis rates? A review of health system factors and approaches associated with thrombolysis administration rates in acute stroke care. Implement Sci. 2016;11(1):51. 27059183 10.1186/s13012-016-0414-6 PMC4825073

[20] McDaid D, Park AL, Iemmi V, Adelaja B, Knapp M. Growth in the use of early intervention for psychosis services: an opportunity to promote recovery amid concerns on health care sustainability; 2016.

[21] Moher D, Liberati A, Tetzlaff J, Altman DG. Preferred reporting items for systematic reviews and meta-analyses: the PRISMA statement. BMJ. 2009;339:b2535. 19622551 10.1136/bmj.b2535 PMC2714657

[22] Popay J, Roberts, H., Snowden, A., Petticrew, M., Arai, L., Rodgers, M., Britten, N., Roen, K., Duffy, S. Guidance on the conduct of narrative synthesis in systematic reviews. Lancaster, UK: Institute of Health Research; 2006.

[23] Geerligs L, Rankin NM, Shepherd HL, Butow P. Hospital-based interventions: a systematic review of staff-reported barriers and facilitators to implementation processes. Implement Sci. 2018;13(1):36. 29475440 10.1186/s13012-018-0726-9 PMC5824580

[24] Braun V, Clarke V. Using thematic analysis in psychology. Qual Res Psychol. 2006;3(2):77–101.

[25] Cranwell K, Polacsek M, McCann TV. Improving mental health service users’ with medical co-morbidity transition between tertiary medical hospital and primary care services: a qualitative study. BMC Health Serv Res. 2016;16(1):302. 27456864 10.1186/s12913-016-1567-3 PMC4960840

[26] Pluye P, Robers, E., Cargo, M., Bartlett, G., O’Cathain, A., Griffiths, F., Boardman, F., Gagnon, M.P., Rousseau, M.C., Robert, E. Proposal: a mixed methods appraisal tool for systematic mixed studies reviews, http://mixedmethodsappraisaltoolpublic.pbworks.com/w/page/24607821/FrontPage; 2011.

[27] Baumann PS, Crespi S, Marion-Veyron R, Solida A, Thonney J, Favrod J, et al. Treatment and early intervention in psychosis program (TIPP-Lausanne): implementation of an early intervention programme for psychosis in Switzerland. Early Interv Psychia. 2013;7(3):322–8. 10.1111/eip.12037 23445318

[28] Brabban A, Dodgson G. What makes early intervention in psychosis services effective? A case study. Early Interv Psychia. 2010;4(4):319–22. 10.1111/j.1751-7893.2010.00169.x 21038749

[29] Essock SM, Kontos N. Implementing assertive community treatment teams. Psychiatr Serv. 1995;46(7):679–83. 7552558 10.1176/ps.46.7.679

[30] Hetrick SE, O’Connor DA, Stavely H, Hughes F, Pennell K, Killackey E, et al. Development of an implementation guide to facilitate the roll-out of early intervention services for psychosis. Early Interv Psychia. 2018;12(6):1100–11. 10.1111/eip.12420 28177191

[31] Iyer S, Jordan G, Macdonald K, Joober R, Malla A. Early intervention for psychosis: A Canadian perspective. J Nerv Ment Dis. 2015;203(5):356–64. 25900548 10.1097/NMD.0000000000000288

[32] Kelly J, Wellman N, Sin J. HEART—the Hounslow Early Active Recovery Team: Implementing an inclusive strength-based model of care for people with early psychosis: practice development. J Psychiatr Ment Health Nurs. 2009;16(6):569–77. 19594681 10.1111/j.1365-2850.2009.01405.x

[33] McGorry PD, Yung AR. Early intervention in psychosis: an overdue reform. Aust N Z J Psychiatry. 2003;37(4):393–8. 12873322 10.1046/j.1440-1614.2003.01192.x

[34] Powell A-L, Hinger C, Marshall-Lee ED, Miller-Roberts T, Phillips K. Implementing coordinated specialty care for first episode psychosis: a review of barriers and solutions. Community Ment Health J. 2021; 57(2): 268–276. 32472286 10.1007/s10597-020-00644-1

[35] Reilly J, Newton R, Dowling R. Implementation of a first presentation psychosis clinical pathway in an area mental health service: the trials of a continuing quality improvement process. Austr Psychiatry. 2007;15(1):14–8. 10.1080/10398560601083027 17464627

[36] Cheng C, Dewa CS, Goering P. Matryoshka project: lessons learned about early intervention in psychosis programme development. Early Interv Psychia. 2011;5(1):64–9. 10.1111/j.1751-7893.2010.00255.x 21272277

[37] Cocchi A, Balbi A, Corlito G, Ditta G, Di Munzio W, Nicotera M, et al. Early intervention in psychosis: a feasibility study financed by the Italian Center on Control of Maladies. Early Interv Psychia. 2015;9(2):163–71. 10.1111/eip.12135 24673891

[38] Durbin J, Selick A, Hierlihy D, Moss S, Cheng C. A first step in system improvement: a survey of Early Psychosis Intervention Programmes in Ontario. Early Interv Psychia. 2016;10(6):485–93. 10.1111/eip.12201 25366518

[39] Ghio L, Natta W, Peruzzo L, Gotelli S, Tibaldi G, Ferrannini L. Process of implementation and development of early psychosis clinical services in Italy: a survey. Early Interv Psychia. 2012; 6(3):341–6. 10.1111/j.1751-7893.2012.00340.x 22309447

[40] Gidugu V, Rogers ES, Gordon C, Elwy AR, Drainoni ML. Client, family, and clinician experiences of Open Dialogue-based services. Psychol Serv. 2021; 18(2):154–163. 31916809 10.1037/ser0000404

[41] Gorrel J, Cornish A, Tennant C, Rosen A, Nash L, McKay D, et al. Changes in early psychosis service provision: A file audit. Aust N Z J Psychiatry. 2004;38(9):687–93. 15324332 10.1080/j.1440-1614.2004.01450.x

[42] Lester H, Birchwood M, Bryan S, England E, Rogers H, Sirvastava N. Development and implementation of early intervention services for young people with psychosis: case study. Br J Psychiatry. 2009;194(5):446–50. 19407276 10.1192/bjp.bp.108.053587

[43] Maric NP, Andric Petrovic S, Rojnic-Kuzman M, Riecher-Rössler A. Implementation of early detection and intervention services for psychosis in Central and Eastern Europe: current status. Early Interv Psychia. 2019;13(5):1283–8. 10.1111/eip.12805 30900823

[44] North CS, Simic Z, Burruss J. Design, implementation, and assessment of a public comprehensive specialty care program for early psychosis. J Psychiatr Pract. 2019;25(2):91–102. 30849057 10.1097/PRA.0000000000000364

[45] Pinfold V, Smith J, Shiers D. Audit of early intervention in psychosis service development in England in 2005. Psychiatr Bull R Coll Psychiatr. 2007;31(1):7–10.

[46] Tiffin PA, Glover G. From commitment to reality: early intervention in psychosis services in England. Early Interv Psychia. 2007;1(1):104–7. 10.1111/j.1751-7893.2007.00004.x 21352114

[47] White DA, Luther L, Bonfils KA, Salyers MP. Essential components of early intervention programs for psychosis: available intervention services in the United States. Schizophr Res. 2015;168(1–2):79–83. 26307427 10.1016/j.schres.2015.08.020

[48] Essock SM, Nossel IR, McNamara K, Bennett ME, Buchanan RW, Kreyenbuhl JA, et al. Practical monitoring of treatment fidelity: examples from a team-based intervention for people with early psychosis. Psychiatr Services. 2015;66(7):674–65. 10.1176/appi.ps.201400531 PMC4490109 25555176

[49] Wisdom JP, Knapik S, Holley MW, Van Bramer J, Sederer LI, Essock SM. Best practices: New York’s outpatient mental health clinic licensing reform: using tracer methodology to improve service quality. Psychiatr Serv. 2012;63(5):418–20. 22549525 10.1176/appi.ps.20120p418

[50] Essock SM, Goldman HH, Hogan MF, Hepburn BM, Sederer L, Dixon LB. State Partnerships for First-Episode Psychosis Services. Psychiatr Serv. 2015;66(7):671–3. 25555092 10.1176/appi.ps.201400117

[51] McDonald K, Ding T, Ker H, Dliwayo TR, Osborn DPJ, Wohland P, et al. Using epidemiological evidence to forecast population need for early treatment programmes in mental health: a generalisable Bayesian prediction methodology applied to and validated for first-episode psychosis in England. Br J Psychiatry. 2021;219(1):383–391. 34475575 10.1192/bjp.2021.18 PMC7611597

[52] Beiser M, Erickson D, Fleming JA, Iacono WG. Establishing the onset of psychotic illness. Am J Psychiatry. 1993;150(9):1349–54. 8352345 10.1176/ajp.150.9.1349

[53] Häfner H, Riecher-Rössler A, Hambrecht M, Maurer K, Meissner S, Schmidtke A, et al. IRAOS: an instrument for the assessment of onset and early course of schizophrenia. Schizophr Res. 1992;6(3):209–23. 1571314 10.1016/0920-9964(92)90004-o

[54] Singh SP, Cooper JE, Fisher HL, Tarrant CJ, Lloyd T, Banjo J, et al. Determining the chronology and components of psychosis onset: the Nottingham Onset Schedule (NOS). Schizophr Res. 2005;80(1):117–30. 15978778 10.1016/j.schres.2005.04.018

[55] Department of Health. The mental health policy implementation guide. London: Department of Health, UK; 2001.

[56] NHS England. Implementing the early intervention in psychosis access and waiting time standard: guidance; 2016.

[57] Birchwood M, Connor C, Lester H, Patterson P, Freemantle N, Marshall M, et al. Reducing duration of untreated psychosis: care pathways to early intervention in psychosis services. Br J Psychiatry. 2013;203(1):58–64. 23703317 10.1192/bjp.bp.112.125500

[58] Maruthappu M, Hasan A, Zeltner T. Enablers and barriers in implementing integrated care. Health Syst Reform. 2015;1(4):250–6. 31519094 10.1080/23288604.2015.1077301

[59] Darker CD, Nicolson GH, Carroll A, Barry JM. The barriers and facilitators to the implementation of National Clinical Programmes in Ireland: using the MRC framework for process evaluations. BMC Health Serv Res. 2018;18(1):733. 30249262 10.1186/s12913-018-3543-6 PMC6154419

[60] Weiner BJ, Mettert KD, Dorsey CN, Nolen EA, Stanick C, Powell BJ, et al. Measuring readiness for implementation: a systematic review of measures’ psychometric and pragmatic properties. Implement Res Pract. 2020;1:2633489520933896. 10.1177/2633489520933896 PMC9924280 37089124

[61] Morris S, Hunter RM, Ramsay AIG, Boaden R, McKevitt C, Perry C, et al. Impact of centralising acute stroke services in English metropolitan areas on mortality and length of hospital stay: difference-in-differences analysis. BMJ. 2014;349:g4757. 25098169 10.1136/bmj.g4757 PMC4122734

[62] Turner S, Ramsay A, Perry C, Boaden R, McKevitt C, Morris S, et al. Lessons for major system change: centralization of stroke services in two metropolitan areas of England. J Health Serv Res Policy. 2016;21(3):156–65. 26811375 10.1177/1355819615626189 PMC4904350

