# Supplementary material for: Early Intervention in Psychosis services: A systematic review and narrative synthesis of the barriers and facilitators to implementation
Source: Eur Psychiatry. 2021 Dec 16;65(1):e2. doi: 10.1192/j.eurpsy.2021.2260 (PMC8792869; doi:10.1192/j.eurpsy.2021.2260)
Supplement: Supplementary file 1 [file S0924933821022604sup001.docx]

*Supplementary Material*

Search strategy

EMBASE 1379 Results

'psychosis'/exp OR 'schizophrenia spectrum disorder'/exp

(schizo* OR psychotic* OR psychosis OR psychoses OR hebephreni* OR oligophreni*):ti,ab

((chronic* OR sever*) Near/5 mental* NEAR/5 (ill* OR disorder*)):ti,ab

#1 OR #2 OR #3

((risk* NEAR/3 schiz*) OR (screen* NEAR/3 schiz*)):ti,ab

((duration OR length) NEAR/3 untreat*):ti,ab

((first OR initial OR primary) NEAR/3 (admission* OR hospital* OR episod* OR breakdown*)):ti,ab

((early OR ‘first episode’ OR ‘first onset’) NEAR/3 (intervent* OR treat* OR recogni* OR detect*)):ti,ab

(delay* NEAR/3 treat*):ti,ab

('(DUP)' OR premorbid* OR prodrom*):ti,ab

#5 OR #6 OR #7 OR #8 OR #9 OR #10

'evaluation study'/exp OR 'qualitative research'/exp OR 'process evaluation'/exp OR 'feasibility study'/exp OR 'case study'/exp

((process OR program* OR systematic) NEAR/3 (evaluation*)):ti,ab

(‘black box’ OR ‘qualitative component*’ OR ‘qualitative aspect*’ OR ‘qualitative approach*’ OR ‘participant observation’ OR simulation OR ‘implementation audit’ OR 'feasibility stud*’):ti,ab

(audit OR audits OR feedback OR ‘proof of concept’ OR ‘survey study’):ti,ab

(((‘semi-structured’ or semistructured or unstructured or informal or ‘in-depth’ or indepth or ‘face-to-face’ or structured or guide) NEAR/3 (interview* or discussion* or questionnaire*))):ti,ab

(‘focus group*’ or qualitative or ethnograph* or fieldwork or ‘field work’ or ‘key informant’):ti,ab

#12 OR #13 OR #14 OR #15 OR #16 OR #17

#4 AND #11 AND #18

'editorial'/exp OR 'erratum'/de OR 'letter'/exp OR 'conference abstract':it OR 'conference review':it

#19 NOT #20

Medline 2120

exp Schizophrenia/ OR exp Psychotic Disorders/

(schizo* OR psychotic* OR psychosis OR psychoses OR hebephreni* OR oligophreni*).ti,ab.

((chronic* OR sever*) adj5 mental* adj5 (ill* OR disorder*)).ti,ab.

or/1-3

((risk* adj3 schiz*) OR (screen* adj3 schiz*)).ti,ab.

((duration OR length) adj3 untreat*).ti,ab.

((first OR initial OR primary) adj3 (admission* OR hospital* OR episod* OR breakdown*)).ti,ab.

((early OR first episode OR first onset) adj3 (intervent* OR treat* OR recogni* OR detect*)).ti,ab.

(delay* adj3 treat*).ti,ab.

(DUP OR premorbid* OR prodrom*).ti,ab.

or/5-10

evaluation study/ OR Qualitative Research/ OR Program Evaluation/ OR Feasibility Studies/ OR Case Reports/

((process OR program* OR systematic) adj3 (evaluation*)).ti,ab.

(black box OR qualitative component* OR qualitative aspect* OR qualitative approach* OR participant observation OR simulation OR implementation audit OR feasibility stud*).ti,ab.

(audit OR audits OR feedback OR proof of concept OR survey study).ti,ab.

(((semi-structured or semistructured or unstructured or informal or in-depth or indepth or face-to-face or structured or guide) adj3 (interview* or discussion* or questionnaire*))).ti,ab.

(focus group* or qualitative or ethnograph* or fieldwork or field work or key informant).ti,ab.

or/12-17

4 AND 11 AND 18

Web of Science 1553

TS =((schizo* OR psychotic* OR psychosis OR psychoses OR hebephreni* OR oligophreni*) OR ((chronic* OR sever*) NEAR/4 mental* NEAR/4 (ill* OR disorder*)))

TS =(((risk* NEAR/2 schiz*) OR (screen* NEAR/2 schiz*)) OR ((duration OR length) NEAR/2 untreat*) OR ((first OR initial OR primary) NEAR/2 (admission* OR hospital* OR episod* OR breakdown*)) OR ((early OR “first episode” OR “first onset”) NEAR/2 (intervent* OR treat* OR recogni* OR detect*)) OR (delay* NEAR/2 treat*) OR (DUP OR premorbid* OR prodrom*))

TS =(((process OR program* OR systematic) NEAR/2 (evaluation*)) OR (“black box” OR “qualitative component*” OR “qualitative aspect*” OR “qualitative approach*” OR “participant observation” OR simulation OR “implementation audit” OR “feasibility stud*”) OR (audit OR audits OR feedback OR “proof of concept” OR “survey study”) OR (((“semi-structured” or semistructured or unstructured or informal or “in-depth” or indepth or “face-to-face” or structured or guide) NEAR/2 (interview* or discussion* or questionnaire*))) OR (“focus group*” or qualitative or ethnograph* or fieldwork or “field work” or “key informant”))

#1 AND #2 AND #3

PsycINFO 1237

DE "Psychosis" OR DE "Acute Psychosis" OR DE "Affective Psychosis" OR DE "Chronic Psychosis" OR DE "Paranoia (Psychosis)" OR DE "Reactive Psychosis" OR DE "Schizophrenia" OR DE "Paranoid Schizophrenia"

TI (schizo* OR psychotic* OR psychosis OR psychoses OR hebephreni* OR oligophreni*) OR AB (schizo* OR psychotic* OR psychosis OR psychoses OR hebephreni* OR oligophreni*)

TI ((chronic* OR sever*) N4 mental* N4 (ill* OR disorder*)) OR AB ((chronic* OR sever*) N4 mental* N4 (ill* OR disorder*))

S1 OR S2 OR S3

TI ((risk* N3 schiz*) OR (screen* N3 schiz*)) OR AB ((risk* N3 schiz*) OR (screen* N3 schiz*))

TI ((duration OR length) N3 untreat*) OR AB ((duration OR length) N3 untreat*)

TI ((first OR initial OR primary) N3 (admission* OR hospital* OR episod* OR breakdown*)) OR AB ((first OR initial OR primary) N3 (admission* OR hospital* OR episod* OR breakdown*))

TI ((early OR “first episode” OR “first onset”) N3 (intervent* OR treat* OR recogni* OR detect*)) OR AB ((early OR “first episode” OR “first onset”) N3 (intervent* OR treat* OR recogni* OR detect*))

TI (delay* N3 treat*) OR AB (delay* N3 treat*)

TI (“DUP” OR premorbid* OR prodrom*) OR AB (“DUP” OR premorbid* OR prodrom*)

S5 OR S6 OR S7 OR S8 OR S9 OR S10

DE "Qualitative Methods"

TI ((process OR program* OR systematic) N2 (evaluation*)) OR AB ((process OR program* OR systematic) N2 (evaluation*))

TI (“black box” OR “qualitative component*” OR “qualitative aspect*” OR “qualitative approach*” OR “participant observation” OR simulation OR “implementation audit” OR “feasibility stud*”) OR AB (“black box” OR “qualitative component*” OR “qualitative aspect*” OR “qualitative approach*” OR “participant observation” OR simulation OR “implementation audit” OR “feasibility stud*”)

TI (audit OR audits OR feedback OR “proof of concept” OR “survey study”) OR AB (audit OR audits OR feedback OR “proof of concept” OR “survey study”)

TI (((“semi-structured” or semistructured or unstructured or informal or “in-depth” or indepth or “face-to-face” or structured or guide) N3 (interview* or discussion* or questionnaire*))) OR AB (((“semi-structured” or semistructured or unstructured or informal or “in-depth” or indepth or “face-to-face” or structured or guide) N3 (interview* or discussion* or questionnaire*)))

TI (“focus group*” or qualitative or ethnograph* or fieldwork or “field work” or “key informant”) OR AB (“focus group*” or qualitative or ethnograph* or fieldwork or “field work” or “key informant”)

S12 OR S13 OR S14 OR S15 OR S16 OR S17

S4 AND S11 AND S18
